# Dysregulated cytokine and growth factor expression in OSSN HIV-1 patients from Botswana with multiple infections

**DOI:** 10.1186/1750-9378-7-S1-P46

**Published:** 2012-04-19

**Authors:** Kenneth O Simbiri, Erle S Robertson

**Affiliations:** 1 Perelman Medical School of the University of Pennsylvania, Philadelphia, PA, USA

## Background

Ocular surface squamous neoplasia (OSSN) is a conjunctival or corneal neoplastic tumor that is becoming prevalent in HIV-1 infected patients. Prior to the HIV pandemic, OSSN was noted to occur predominantly in the elderly for whom it is the third most common oculoorbital tumor after melanoma and lymphoma. In Africa OSSN is becoming more common, more aggressive, and affects young people, especially females. In parallel with the dramatic increase of HIV in Africa, several countries have noted a sharp rise in the incidence of OSSN in HIV infected individuals such that OSSN is currently the most common ocular tumor among adults. The underlying cause of this cancer in HIV-infected patients from Botswana is not well defined.

## Method

Diluted sera from OSSN, pterygia, and control samples were used in Ray Biotech Assay kit for determination of expression of several cytokines and growth factors. We extracted RNA from tissue samples and used designed type specific primers for cytokines and growth factors to analyze expression. The samples were further analyzed for the expression of other pathogens using pyrosequencing technology.

## Results

Cytokine array results from OSSN and pterygia cases indicated expression of some inflammatory cytokines and growth factors associated with tumor development and growth. Further, quantitative RT-PCR showed the expression of similar inflammatory cytokines and growth factors by a panel of OSSN and pterygia tissues. The expression of the factors were not different in the two conditions of OSSN and pterygia respectively. Additional analysis utilizing pyrosequencing technique identified a number of bacterial, viral, parasitic, and fungal sequences in the patient samples.

## Conclusion

We identified anti-inflammatory cytokines and growth factors associated with cancer pathogenesis in OSSN and pterygia tissues. We also showed sequences of bacterial, parasitic, fungal, and other viral pathogens in the samples that may contribute to immunosuppression. Further studies are necessary to characterize the molecular mechanisms associated with cytokines and growth factors elicited by oncogenic viral proteins and the development of OSSN. Studies to elucidate the significance of other infectious pathogens in OSSN pathology will be necessary.

